# Anastomosing hemangioma of the ovary – a comprehensive review of this rare ovarian entity

**DOI:** 10.2478/raon-2024-0050

**Published:** 2024-09-15

**Authors:** Sebastjan Merlo, Gregor Vivod, Barbara Gazic, Nina Kovacevic

**Affiliations:** Department of Gynecological Oncology, Institute of Oncology Ljubljana, Ljubljana, Slovenia; Faculty of Medicine, University of Ljubljana, Ljubljana, Slovenia; Faculty of Health Care Angele Boskin, Jesenice, Slovenia; Department of Pathology, Institute of Oncology Ljubljana, Ljubljana, Slovenia

**Keywords:** anastomosing hemangioma, ovary, ovarian hemangioma, urogenital tract

## Abstract

**Background:**

Anastomosing hemangioma of the ovary is a rare vascular tumor that predominantly affects middle-aged women. Despite its benign nature, its histological appearance can mimic aggressive vascular lesions, posing diagnostic challenges. This review aims to provide an overview of this uncommon entity.

**Methods:**

The PubMed and Scopus databases were searched for relevant articles published in English. Information on all retrieved cases was extracted and reviewed in detail.

**Results:**

We found 33 cases with relevant details of anastomosing heamangioma of the ovary. Despite the small number of cases we found, our study demonstrated the importance of an accurate hystopathological evaluation.

**Conclusions:**

Although the preliminary imaging and initial microscopic features may appear alarming, careful microscopic examination reveals benign behavior. There is a need to raise awareness of this unusual and rare entity to improve morphologic recognition and avoid misdiagnosis that could lead to unnecessary treatment or patient anxiety.

## Introduction

Anastomosing hemangioma is a rare benign vascular tumor reported to occur in the kidney, testis, paravertebral soft tissue, gastrointestinal tract, liver, and in rare instances, in the ovary.^1,2,3,4^ It was first described in 2009 by Montgomery and Epstein.^5^ Since then, only a limited number of cases have been reported in the literature, contributing to its diagnostic uncertainty. Most cases of anastomosing hemangioma of the ovary presents as a solitary, well-circumscribed mass or is incidental findings.^1^ Occasionally, ovarian anastomosing hemangiomas have been associated with ascites and elevated serum CA 125 levels raising concern for an epithelial malignancy. Ovarian hemangiomas can be mistaken for malignant ovarian tumors due to their appearance on imaging studies. Appropriate preoperative diagnostics, including ultrasound, MRI, and sometimes CT scans, are essential to differentiate hemangiomas from more serious conditions such as ovarian cancer, which would require a different surgical approach and postoperative management.^6,7^

Histologically, anastomosing hemangioma is characterized by anastomosing sinusoidal capillary sized blood vessels lined by bland endothelial cells with hobnail appearance, in a hyalinized and edematous stroma. The cells may show minimal atypia, which can be mistaken for low-grade angiosarcoma.^7^ Immunohistochemical staining may reveal positivity for endothelial markers such as CD31 and CD34, aiding in its diagnosis.^8^

We reviewed the current and relevant literature on anastomosing hemangiomas of the ovary to increase awareness of this rare entity. Prior to our review, only 33 cases of anastomosing hemangioma of the ovary have been reported in the English-language literature.^1,3,6,7,8,9,10,11,12,13,14,15^ Most of reviews are presented as case reports or small case series, and the largest series includes 12 cases of anastomosing hemangioma of the ovary.^9^ To add to the literature, we present and describe our case of anastomosing hemangioma of the ovary.

## Materials and methods

### Search strategy

The PubMed database was searched for relevant articles published in English between January 1990 and December 2023. The search strategy included the following terms: (Hemangioma[mesh] OR Hemangioma*[tiab]) AND (anastomosing Hemangioma*[tiab] OR anastomosing[tiab] OR anastomos*[tiab]) AND (Ovary[mesh] OR Ovary[tiab] OR ovaries[tiab] OR ovarina[tiab] OR Ovarian Neoplasms[mesh] OR Ovarian Neoplasms[tiab] OR Cancer of Ovary[tiab] OR Cancer of the Ovary[tiab] OR Ovarian Cancer*[tiab] OR Ovary Cancer*[tiab] OR Ovary Neoplasms[tiab] OR ovarian malignancy[tiab] OR ovarian tumor*[tiab] OR Ovarian Carcinoma[tiab]) AND 1990/01/01:2024/01/01[Date - Publication]. The Scopus database was also searched. The references of all relevant reviews found were also examined to avoid omitting qualified studies. In addition, references to related articles were searched to identify studies that might meet the criteria. Evaluation of each article was conducted independently by three reviewers (S.M., G.V. and N.K.).

### Inclusion criteria

The inclusion criteria were as follows: Women with a histopathological diagnosis of anastomosing hemangioma of the ovary; all published retrospective small studies and case reports containing patient-relevant information; clinical presentation, size of the hemangioma; stromal lutenization. In the case of duplicates in the literature, the most recent and comprehensive articles were selected. We also included the case report by Metodiev *et al*. in which the abstract was written in English.^12^

### Exclusion criteria

The articles were excluded for one of the following reasons: Articles not specifying the type of ovarian tumor and articles not in English language.

### Data extraction

Study information that were extract and reviewed in detail: Patient age at diagnosis, tumor size and location, histopathological type, clinical presentation, and presence of stromal luteinization.

## Results

### A new case

A 60 years old woman was sent to Department of Gynecological Oncology, Institute of Oncology Ljubljana with a suspected left-sided ovarian mass. Vaginal ultrasound showed a mixed cystic and solid appearance. The largest diameter of the mass was 50 millimeters. Family history was negative for malignancy. Her medical history was unremarkable. The tumor markers CA 125, CEA, HE4, CA 19-9, CA 72-4 and CA 15-3 were all negative. She underwent laparoscopic bilateral salpingo-oophorectomy. Left ovary was partly fragmented. In one of the fragments, there was a well circumscribed lesion, measuring 25 mm in diameter, with yellow-brown, spongy appearance was present. Microscopic examination of H&E-stained slides revealed well-demarcated vascular proliferation, composed mostly of capillary-sized blood vessels with an anastomosing growth pattern and some larger vessels of medium size. Vessels lining was composed of a single layer of endothelial cells without cytologic atypia or mitotic figures (Figure 2). The lesion was surrounded by luteinized ovarian stroma. Immunohistochemically the tumor cells were positive for CD31, CD34 and ERG (Figure 3).

**FIGURE 1.**
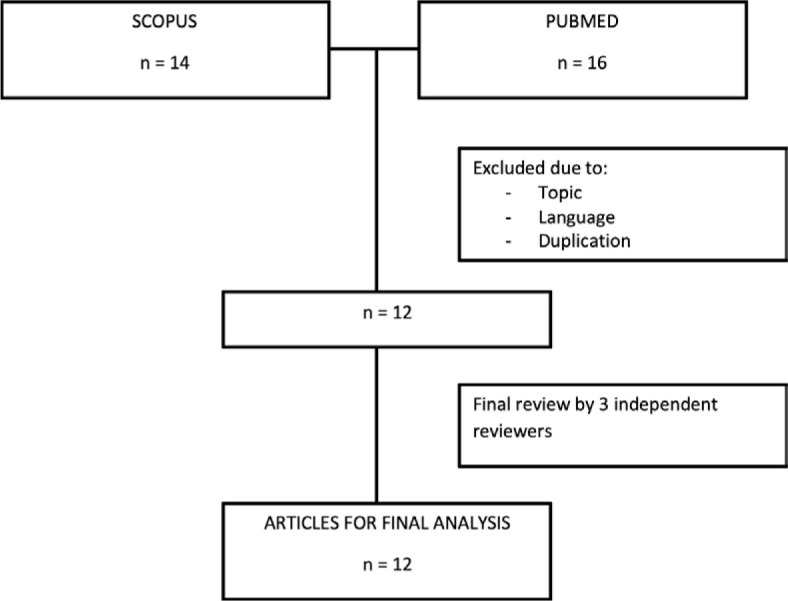
Literature review flowchart.

**FIGURE 2.**
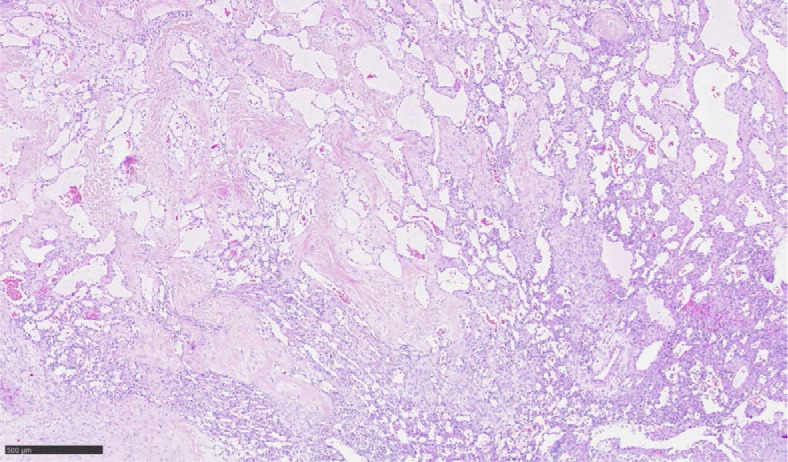
HE: Anastomosing sinusoidal-like vessels lined by endothelial cells without cytologic atypia.

**FIGURE 3.**
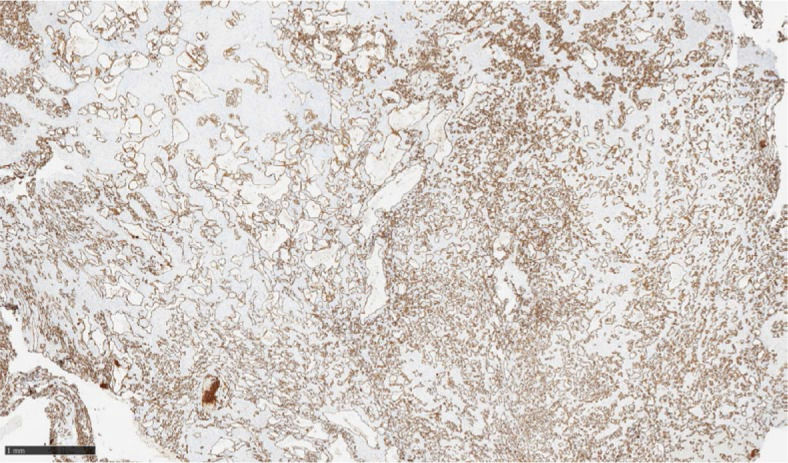
CD 31 highlights endothelial cells lining numerous vessels.

### Review of literature

A flowchart showing the phases of the search strategy is shown in Figure 1. Our search in the Scopus and PubMed databases initially returned 30 results. After the initial screening, 18 articles were excluded due to duplicates and non-English language. Of the remaining 12 articles, the titles and abstracts were screened by reviewers and were classified as relevant and were subjected to a full text and literature review. For the final analysis all 12 papers with relevant details were selected.

The mean age of the 32 women diagnosed with anastomosing hemangioma of the ovary was 59.2 years. In 18 women tumor was incidental finding, three women presented with elevated CA 125 serum level and ascites. Only ascites was present in one patient and five women have ultrasound detected ovarian mass. The mean maximum diameter of tumor was 20.6 mm. The detailed data of the patients and the tumor characteristics are listed in Table 1.

**TABLE 1.** Published cases of anastomosing hemangiomas of the ovary in the English literature

**No**	**Author**	**Age**	**Clinical presentation**	**Size (mm)**	**Stromal luteinization**
1	Metodiew *et al*.^12^	70	NA	7	Yes
2	Kryvenko *et al*.^3^	70	Incidental	2	No
3	Kryvenko *et al*.^3^	49	Incidental	1	No
4	Kryvenko *et al*.^3^	77	Incidental	11	Yes
5	O’Neill *et al*.^10^	NA	NA	NA	NA
6	O’Neill *et al*.^10^	NA	NA	NA	NA
7	O’Neill *et al*.^10^	NA	NA	NA	NA
8	O’Neill *et al*.^10^	NA	NA	NA	NA
9	Dundr *et al*.^6^	66	Incidental	5	Yes
10	Dundr *et al*.^6^	43	Incidental	13	Yes
11	Dundr *et al*.^6^	69	Incidental	15	Yes
12	Dundr *et al*.^6^	81	Incidental	35	Yes
13	Dundr *et al*.^6^	68	Ascites, elevated CA 125	35	Yes
14	Dundr *et al*.^6^	69	Mass	12	Yes
15	Gunduz *et al*.^8^	62	Incidental	90	Yes
16	Subbarayan *et al*.^7^	50	Ascites	30	Yes
17	Rezk *et al*.^1^	60	Ascites, elevated CA 125	65	Yes
18	Stewart and Salfinger^11^	48	Incidental	8	Yes
19	McHenry and Buza^9^	55	Mass	12	Yes
20	McHenry and Buza^9^	62	Mass	10	Yes
21	McHenry and Buza^9^	67	Incidental	5	Yes
22	McHenry and Buza^9^	76	Incidental	7	Yes
23	McHenry and Buza^9^	58	Incidental	8	Yes
24	McHenry and Buza^9^	53	Mass	6	Yes
25	McHenry and Buza^9^	73	Incidental	4	Yes
26	McHenry and Buza^9^	65	Incidental	10	Yes
27	McHenry and Buza^9^	50	Incidental	9	Yes
28	McHenry and Buza^9^	69	Incidental	6	No
29	McHenry and Buza^9^	63	Incidental	3	No
30	McHenry and Buza^9^	55	Incidental	2	No
31	Wang *et al*.^13^	28	Mass	40	No
32	Wu *et al*.^14^	26	Mass, ascites, elevated CA 125	46	NA
33	Jha *et al*.^15^	35	Mass	100	Yes

NA = not available

## Discussion

The definition of anastomosing hemangioma was established in 2009.^5^ The most likely theory that explaining the pathogenesis of this phenomenom characterizes anastomosing hemangioma of ovary as behaving like enlarging follicles that cause pressure on the neighboring tissue and lead to the development of luteinized stromal cells.^6^

In our review of literature, we found 33 cases of anastomosing hemangiomas of the ovary reported in the English literature. In our case the patient was 60 years old, while the mean age in the previous literature was 52.9 years (range from 26 to 81) (Table 1). The tumor size ranged from 1 millimeter to 100 millimeters, with the mean size being 20.6 millimeters. In our case tumor size was 50 millimeters.

Anastomosing hemangioma of the ovary typically affects women in their middle age, although cases have been reported across a wide age range.^6^ Clinically, patients may present with nonspecific symptoms such as abdominal pain, discomfort, or palpable pelvic mass. However, due to its rarity and lack of specific clinical features, anastomosing hemangioma is often an incidental finding on imaging studies or during surgical exploration for unrelated conditions. The absence of pathognomonic symptoms underscores the importance of histopathological evaluation for definitive diagnosis.^16^ Radiological imaging, including ultrasound and MRI, may demonstrate a hypervascular mass, although definitive diagnosis often relies on histological examination following surgical excision.^10^ In our case, too, the ovarian mass was an incidental finding with no specific clinical signs. Tumor markers were all negative and the ultrasound results were non-specific. The final histologic findings were decisive for the final diagnosis.

Stromal luteinization as a unique feature of ovarian anastomosing hemangiomas was reported in 22 of 28 cases (78.6%), and in 5 cases this data was not available (Table 1). Some of the prior cases presented association between anastomosing hemangioma of ovary with ascites and elevated serum CA 125, mimicking an epithelial ovarian malignancy.^1,6,7^ These findings were not observed in our experience.

Particular histologic characteristics are described as non-lobular proliferation of anastomosing capillary sized vessels with sinusoidal-like arrangements resembling the red pulp of the spleen, with vessels lined by bland endothelial cells.^6^ The lack of atypical endothelial cells and mitotic figures helps to differentiate anastomosing hemangiomas from its malignant counterpart, angiosarcoma.^17^ Evaluation of the entire anastomosing hemangioma lesion, when possible, reveals that its growth is limited, lacking a broadly infiltrative pattern. The differential diagnosis of anastomosing hemangioma also includes capillary hemangioma, cavernous hemangioma, hemangioendothelioma, epithelioid hemangioma, and non-neoplastic vascular proliferations.^9^

Surgical excision remains the cornerstone of management for anastomosing hemangioma of the ovary. Complete resection is curative in the majority of cases, with favorable long-term outcomes. Management decisions should be individualized based on factors such as tumor size, location, and patient preferences, with a focus on preserving ovarian function and fertility when feasible. Overall, the prognosis of anastomosing hemangioma of the ovary is excellent following complete surgical resection. Recurrence rates are low, particularly with adequate surgical margins. However, cases of recurrence have been reported, highlighting the importance of vigilant long-term surveillance.^18,19^

Continued collaboration among multidisciplinary teams is essential to advance our understanding and improve outcomes for patients with this rare ovarian tumor. Although the preliminary imaging and initial microscopic features may appear alarming, careful microscopic examination reveals benign behaviour.^10^ There is a need to raise awareness of this unusual and rare entity to improve morphologic recognition and avoid misdiagnosis that could lead to unnecessary treatment or patient anxiety.

## Conclusions

In conclusion, anastomosing hemangioma of the ovary is a rare vascular tumor that poses diagnostic challenges due to its histological resemblance to more aggressive lesions. Despite its benign nature, accurate diagnosis is essential to avoid unnecessary interventions and ensure appropriate management. Long-term follow-up is crucial due to reported cases of recurrence following incomplete resection. Further research is needed to better understand the pathogenesis of this rare ovarian tumor and to optimize diagnostic and therapeutic approaches. Increased awareness among clinicians and pathologists is essential for timely recognition and management of anastomosing hemangioma of the ovary, ultimately improving patient outcomes.
